# Cyclosporin A ameliorates cerebral oxidative metabolism and infarct size in the endothelin-1 rat model of transient cerebral ischaemia

**DOI:** 10.1038/s41598-019-40245-x

**Published:** 2019-03-06

**Authors:** Axel Forsse, Troels Halfeld Nielsen, Kevin Heebøll Nygaard, Carl-Henrik Nordström, Jan Bert Gramsbergen, Frantz Rom Poulsen

**Affiliations:** 1 Department of Neurosurgery, Odense University Hospital, Odense, Denmark; 2 BRIDGE - Brain Research - Inter-Disciplinary Guided Excellence, Department of Clinical Research, University of Southern Denmark, Odense, Denmark; 3 Institute of Molecular Medicine, University of Southern Denmark, Odense, Denmark

## Abstract

Cerebral microdialysis can be used to detect mitochondrial dysfunction, a potential target of neuroprotective treatment. Cyclosporin A (CsA) is a mitochondrial stabiliser that in a recent clinical stroke trial showed protective potential in patients with successful recanalisation. To investigate specific metabolic effects of CsA during reperfusion, and hypothesising that microdialysis values can be used as a proxy outcome measure, we assessed the temporal patterns of cerebral energy substrates related to oxidative metabolism in a model of transient focal ischaemia. Transient ischaemia was induced by intracerebral microinjection of endothelin-1 (150 pmol/15 µL) through stereotaxically implanted guide cannulas in awake, freely moving rats. This was immediately followed by an intravenous injection of CsA (NeuroSTAT; 15 mg/kg) or placebo solution during continuous microdialysis monitoring. After reperfusion, the lactate/pyruvate ratio (LPR) was significantly lower in the CsA group vs placebo (*n* = 17, 60.6 ± 24.3%, *p* = 0.013). Total and striatal infarct volumes (mm^3^) were reduced in the treatment group (*n* = 31, 61.8 ± 6.0 vs 80.6 ± 6.7, *p* = 0.047 and 29.9 ± 3.5 vs 41.5 ± 3.9, *p* = 0.033). CsA treatment thus ameliorated cerebral reperfusion metabolism and infarct size. Cerebral microdialysis may be useful in evaluating putative neuroprotectants in ischaemic stroke.

## Introduction

For several decades it has been recognised that mitochondrial dysfunction, characterised by impaired oxidative metabolism in spite of ample oxygen and substrate delivery, occurs after transient cerebral ischaemia^1,2^. Cerebral mitochondrial dysfunction can be identified and distinguished from cerebral ischaemia by analysing the patterns of energy metabolites obtained by microdialysis under experimental or clinical conditions^3–6^. Microdialysis is currently the only effective means of continuously monitoring cerebral metabolism in neurocritical patients, and it is used to detect secondary injury and to optimise treatment^7^.

A neuroprotective effect of the immunosuppressing drug cyclosporin A (CsA) in experimental transient cerebral ischaemia was first described by Shiga *et al*.^8^. The extent of this effect varies between studies^9–17^, however, and the results under clinical conditions have been equivocal. In a recent prospective randomised clinical study of 127 patients, no significant decrease of 30-day infarct volume was found when comparing CsA to placebo in ischaemic stroke patients eligible for thrombolysis^18,19^. However, the infarct volume was significantly reduced in the subset of patients who had successful recanalisation after proximal cerebral artery occlusion. These results imply that a positive treatment effect of CsA may be dependent on reperfusion and subsequent mitochondrial dysfunction rather than ischaemia per se.

In the current study, standard clinical cerebral microdialysis was used in combination with the previously described endothelin-1 (ET-1) model of focal transient ischaemia^20–22^. Contrary to the standard model of middle cerebral artery occlusion (MCAO), this design allows continuous monitoring in awake, freely moving rats, and effectively avoids the potential confounding effects of anaesthetics^23^.

The aim of the study was to (1) assess the temporal patterns of cerebral energy metabolites associated with mitochondrial dysfunction in the ET-1 rat model of transient focal ischaemia, (2) investigate whether intravenous cyclosporin A improved cerebral oxidative metabolism after reperfusion, and (3) investigate if such an effect correlated with 24-hour infarct volumes.

## Methods and Materials

### Animals and experimental model

**Figure 1:**
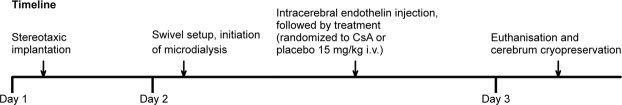
Experiment timeline. Schematic timeline of the 3-day experiment reiterated with two animals per trial. On Day 1, 34 adult Sprague Dawley rats were stereotaxically implanted with two cerebral guides in the left hemisphere and a femoral vein access. On Day 2, the awake and freely moving rats were equipped with microinjection and microdialysis probes and attached to a balanced swivel. Continuous cerebral microdialysis was commenced, and dialysates were analysed every 15 min. When baseline values had stabilised, focal transient ischaemia was induced through intracerebral ET-1 microinjection followed by a randomised i.v. dose of CsA (15 mg/kg NeuroSTAT) or placebo solution. Microdialysis was continued for 5 hours, and after 24 hours rats were sacrificed for histological infarct volume estimation. CsA = cyclosporin A, ET-1 = endothelin-1.

The experiments were conducted from October 2016 to March 2017 at the animal facility of the Institute of Molecular Medicine, University of Southern Denmark, Odense. All experiments were performed in accordance with the Danish Animal Experiment Inspectorate under the Ministry of Food and Agriculture (licence no. 2012-DY-2934- 00008). The study was designed as a reiterated three-day procedure (Fig. 1) with two test animals per trial (treatment and vehicle). Male Sprague Dawley rats (Taconic Biosciences A/S Ejby, Denmark) weighing on average 262 g (range 198–344) and with mean age 7.5 weeks (range 6–9) were individually housed with cage enrichment in a 12-hour light/dark cycle with free access to food and water. This article is written in accordance with the ARRIVE guidelines.

On Day 1, the rats were anaesthetised with a subcutaneous injection of 3 ml/kg Hypnorm/Dormicum (Hypnorm: 0.315 mg/mL fentanyl and 10 mg/mL fluanisone, Janssen Pharmaceuticals, Beerse, Belgium; Dormicum: 5 mg/mL midazolam, Hoffmann-La Roche, Basel, Switzerland) and maintained with a supplemental dose of 1 ml/kg every 40 minutes. Lidocaine (20 mg/ml, Farmaplus AS, Oslo, Norway) was used as a local anaesthetic. Under the microscope (Leica WILD M680), rats were implanted with a heparinised femoral vein catheter (Micro-renatane MRE-033, Braintree/AgnTho’s, Stockholm, Sweden) tunnelled subcutaneously to a cranial midline incision. Using a stereotaxic frame (KOPF instruments, Tujunga, US), two 4 mm microdialysis guides (Brainlink, Groningen, Netherlands) were implanted in the left cerebral hemisphere; one adjacent to the proximal MCA in the piriform cortex (for ET-1 injection), and one in the ipsilateral striatum (for microdialysis). The stereotaxic coordinates relative to bregma, with the nose bar at −3.9 mm (according to the atlas of Paxinos and Watson, 1986), were:

Guide cannula for ET-1 injection: A + 0.9 mm; L 5.2 mm; V −4.6 mm

Guide cannula for microdialysis probe: A + 0.5 mm; L 2.5 mm; V −3.2 mm

The fused silica tubing of a microdialysis probe, from which the membrane was removed, was used as ET-1 probe. After insertion of the injection probe, the tip ended 3.0 mm below the guide cannula, 7.6 mm ventral to bregma.

The guides were fixed to the skull by a glass ionomer luting cement (GC Fuji plus capsule, GC corporation, Tokyo, Japan) with a slotted screw for head block tethering (Instech labs Inc., Plymouth Meeting, PA, USA) to connect the tubing to a dual channel microdialysis swivel (AgnTho’s AB, Stockholm, Sweden) and the tunnelled femoral vein catheter. Body temperature was controlled throughout surgery with a thermostatically regulated heating pad at 37.5 °C (Bosch CTKI3, München, Germany). Postoperative analgesia was achieved through a slow release oral formulation of 0.4 mg/kg buprenorphine (Temgesic 0.2 mg sublingual tab., RB Pharmaceuticals, Slough, UK). 5 ml NaCl was injected subcutaneously to secure post-operative rehydration.

### Experimental Design and Drug Administration

On Day 2, the microdialysis probe (50 kDa, 3 mm membrane, BrainLink, Groningen, the Netherlands) and the ET-1 injection probe, connected with FEP-tubing (1.2 mL/10 cm AgnTho’s AB, Stockholm, Sweden), were inserted and connected to the swivel and monitoring equipment under short-acting isoflurane (Baxter A/S, Allerød, Denmark) anaesthesia. A 100 µL Hamilton syringe containing ET-1 diluted in sterile Ringer’s solution (147 mM NaCl, 4 mM KCl, 1.1 mM CaCl_2_, 1.0 mM MgCl_2_) was connected to the tubing of the ET cannula for manual microinfusion of ET-1. The dialysis probe was perfused with sterile Ringer’s solution using a syringe pump (22 Harvard Apparatus, Inc., Holliston, USA) at a flow rate of 1.0 µL/min. The microdialysis outlet was connected to a sample fraction collector (CMA 142, Stockholm, Sweden) programmed to shift vial every 15 minutes. All connections were made using the aforementioned FEP-tubing and 0.38 mm IDEX silicon. The samples were analysed continuously for glucose, pyruvate and lactate using an ISCUSflex analyser (MDialysis, Stockholm, Sweden) set to sensitivity intervals relevant to the measured concentrations (low linearity).

When a stable baseline was achieved (approx. 3 hours), focal transient ischaemia was induced using a reiterated regimen of intracerebral ET-1 injections of 6 + 3 + 3 + 3 µl (10 pmol/µl, dissolved in sterile Ringer’s solution, stored in 50 µl aliquots at −20 °C; Sigma-Aldrich Denmark A/S, Copenhagen) with 5 min intervals to produce a mean 30 min period of vascular constriction detected by a minimum glucose drop of 50% in concomitant ipsilateral microdialysis. (The optimal dosing regimen was established in pilot studies, where doses previously used by Gramsbergen *et al*.^21^ were modified to minimise permanent occlusion while maximising sufficient ischaemia time). Immediately after ET-1 injection, a randomised blinded intravenous administration of CsA or vehicle (15 mg/kg, 0.5 mg/ml NeuroSTAT and NeuroSTAT-placebo solution, NeuroVive, Lund, Sweden) was commenced at the infusion rate of 0.1 ml/min (maximal tolerated effective single dose – see section 3.3). The microdialysis sampling was continued for at least 5 hours after the insult.

On Day 3 (24 hours after transient ischaemia), the rats were euthanised using barbiturate injection (pentobarbital 200 mg/ml with lidocaine hydrochloride 20 mg/ml, Glostrup Apotek, Denmark) and then decapitated. The brains were rapidly removed and frozen using high pressure CO_2_ and stored in −80 °C until cryostat sectioning, Nissl staining (toluidine blue) and histopathological examination.

### Randomisation and blinding

The experiment was conducted in series using two rats per trial. All surgical and non-surgical procedures were identical until randomisation to treatment or placebo. Randomisation and blinding of treatment versus placebo were performed using unlabelled vials with content unknown to the investigator (numbered 1 and 2). The animals were numbered with a tail marking and paired with treatment number. Unblinding was performed at the end of the experimental procedures. The infarct volume was estimated by investigators blinded to the treatment group assignment.

Altogether, 34 animals were randomised. One animal died after randomisation, and transient ischaemia was not achieved in two animals as evaluated by continuous real-time analysis of cerebral microdialysates of glucose concentrations in the affected cerebral volume (all three randomised to placebo group). Accordingly, infarct volume was evaluated in 17 CsA animals and 14 placebo animals. Due to technical problems with microdialysis perfusion flow rendering further analyses impossible (12 rats), as well as exclusion of animals displaying permanent MCA occlusion as defined by incomplete normalisation of cerebral glucose in real-time continuous cerebral microdialysis (2 rats), the complete biochemical analysis was performed in 10 CsA animals and 7 placebo animals.

### Infarct volumetry

The whole rat forebrain was sectioned coronally into 30 μm slices in three parallel series (Leica CM 3050S cryostat) that were then placed on microscope slides. Two series were kept for reference, and one series was used for infarct volume (IFV) estimation and stained with toluidine blue^24^. Correct placement of probes was confirmed. Infarct volume was estimated using the Computer Assisted Stereological Test (CAST) Grid System (Olympus, Denmark) and the Cavalieri principle for volume estimation as described by Lambertsen *et al*.^25^ (standardised from 3.12 mm anterior and 3.12 mm posterior to the anterior commissure).

### Statistics

All results are reported as mean ± SEM unless otherwise stated. Metabolite concentrations were visualised in timeline graphs. Means were calculated with values clustered at 15 min intervals. Lactate/pyruvate ratios (LPR) computed separately for specimens were normalised to baseline (i.e. changes of LPR are expressed as mean percentage of baseline values) in ICUpilot (v 2.0, MDialysis software, Sweden). Outliers were not cleared except in cases of documented instrumental failure and unintended permanent MCA occlusion. For statistical comparison of LPR (normally distributed in QQ-plot), a mixed effects linear regression model for repeated measurements was used with time and group as random effect and individual specimen as fixed effect. When an overall treatment effect was found, post hoc linear combinations of estimators were used to generate P values at the selected time interval, and regression diagnostics confirmed normally distributed residuals. Infarct volumes were analysed with regard to striatal and total (striatal + cortical) infarction and grouped according to treatment. Student’s t-test was used to analyse difference in means after confirming normal distribution using the QQ-plot technique. A P value below 0.05 was considered significant. Sample size estimation for comparing metabolite concentrations was based on variance from a pilot study (*n* = 10, SD 3.8), with a power of 80% and significance level at 5% and giving 7–8 rats per group based on a minimum treatment effect of 20% (STATA v 14.1, StataCorp., US).

## Results

Table 1 presents the original mean baseline concentrations of the energy metabolites glucose, lactate, pyruvate and the lactate/pyruvate ratio along with their mean percentage change after reperfusion compared to the normalised baseline.

**Table 1:** Mean metabolite concentrations ± SEM of continuous cerebral microdialysis in the endothelin-1 rat model for animals treated with cyclosporin A (CsA) or placebo. Baseline concentrations of glucose, lactate, pyruvate and lactate/pyruvate ratio (LPR) and their mean percentage change from normalised baseline ± SEM (mixed effects regression model analysis) after reperfusion (time period 3–5 hours in Fig. 2). Dialysis perfusion flowrate −1 µl/min, 3 mm membrane length.

Baseline	Glucose mmol/L	Lactate mmol/L	Pyruvate µmol/L	LPR ratio
CsA (*n* = 10)	0.30 ± 0.04	0.21 ± 0.02	27.5 ± 3.8	8.2 ± 0.8
Placebo (*n* = 7)	0.34 ± 0.05	0.19 ± 0.02	30.8 ± 5.2	7.0 ± 1.1
**After reperfusion**	**Glucose % of baseline**	**Lactate % of baseline**	**Pyruvate % of baseline**	**LPR % of baseline**
CsA (*n* = 10)	113.7 ± 18.4	125.4 ± 24.5	100.5 ± 15.9	126.2 ± 24.3*
Placebo (*n* = 7)	106.8 ± 14.3	168.9 ± 18.5	92.8 ± 12.0	186.8 ± 18.4

*Statistical significance compared to placebo (*p* = 0.013).

**Figure 2:**
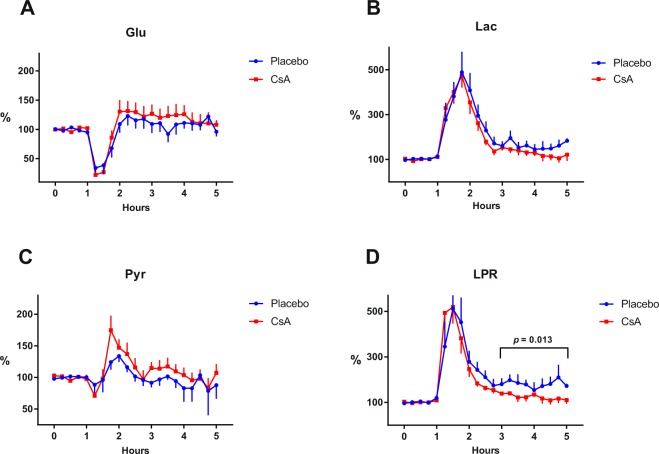
Temporal patterns of microdialysis metabolite concentrations – treatment vs placebo. Timeline graphs with normalised baselines showing the dynamic changes of brain interstitial mean (±SEM) concentrations of main intermediate metabolites of the redox-state in CsA (*n* = 10) and placebo (*n* = 7) specimens. At 1 hour, transient cerebral ischaemia was induced through intracerebral microinjection of ET-1. (**A**) Glucose levels showed similar patterns of transient ischaemia/reperfusion in both groups. (**B**) Lactate levels were very similar in the two groups, with low variance and a tendency to lower post-ischaemic values in the treatment group. (**C**) Pyruvate levels with the characteristic dip and overshoot pattern of this model, with CsA-treated animals showing slightly higher values. (**D**) The primary outcome variable LPR showed a significant point difference of 60.6 ± 24.3% at 3–5 hours (*p* = 0.013, mixed effect regression analysis). CsA = cyclosporin A, ET-1 = endothelin-1, LPR = lactate/pyruvate ratio.

Figure 2 illustrates the ET-1 induced changes of glucose, lactate, pyruvate and LPR in the rat striatum for treatment and placebo groups. Immediately after ET-1 injection (Fig. 2, 1 hour time point), a nearly identical, marked decrease in glucose level was seen, confirming the reliability of the lesioning technique. The subsequent increase in glucose after approximately 30 min, indicating return of blood flow, was similar in both groups.

Lactate levels increased markedly in both groups shortly after induction of ischaemia. The subsequent decrease of lactate toward baseline and the simultaneous increase in pyruvate concentration was more pronounced in the CsA treated animals. As a result, LPR decreased more in the CsA group, almost reaching normal baseline level (Fig. 2D). The mean 60.6 ± 24.3% point difference in LPR between the two groups (CsA 126.2 ± 24.3% vs controls 186.8 ± 18.4%) in the 3–5 hours after induction of ischaemia was significant (*p* = 0.013).

**Figure 3:**
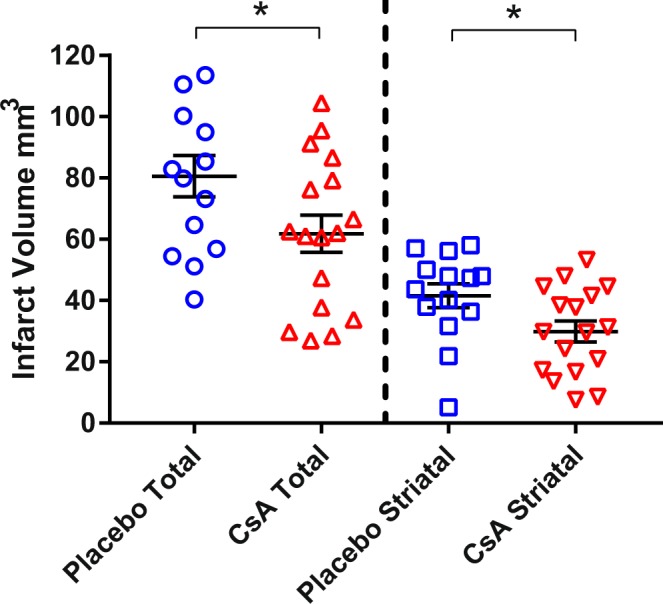
24-hour total and striatal infarct volumes – treatment vs placebo. Scatterplot with mean (±SEM) infarct volumes (mm^3^ in CsA (*n* = 17) and placebo (*n* = 14) groups. The two left columns show total infarct volume, the two right columns show the striatal infarct volumes alone. The infarct volume difference is significant for both total volume (61.8 ± 6.0 vs 80.6 ± 6.7, *p* = 0.047) and striatal volume (29.9 ± 3.5 vs 41.5 ± 3.9, *p* = 0.033).

As shown in Fig. 3, the decreases in the total and striatal 24-hour infarct volumes (mm^3^) were statistically significant in the 17 animals treated with CsA compared to the 14 placebo animals (total volume: 61.8 ± 6.0 vs 80.6 ± 6.7, *p* = 0.047 and striatal volume: 29.9 ± 3.5 vs 41.5 ± 3.9, *p* = 0.033).

**Figure 4:**
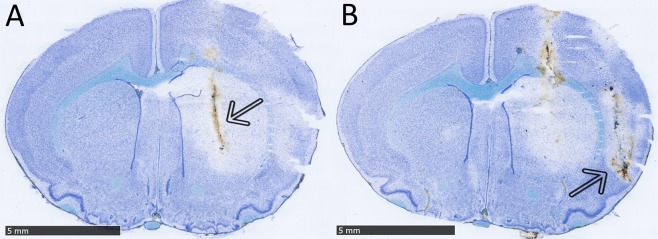
Representative micrographs of toluidine-stained rat brain sections – microdialysis and endothelin-1 probe placement. Representative toluidine blue-stained 30 µm rat brain sections with visible striatal and cortical infarcts and 5 mm scale bars. Arrows indicate the location of the microdialysis probe canal in the centre striatum (**A**) and the endothelin-1 probe canal in the piriform cortex (**B**).

In each specimen, the stereotaxic placement of the microdialysis probe and the ET-1 cannula was confirmed in the histologic sections with the intended 0.4 mm AP offset (Fig. 4, see method section for coordinates).

## Discussion

Relating to the treatment of acute cerebrovascular disease, this study links the potential neuroprotectant CsA^26^ to the proxy outcome measure of a widely used neurocritical monitoring method. It describes the effect of CsA treatment on oxidative metabolism and mitochondrial function in the ET-1 ischaemia-reperfusion model.

The results of this study are of direct clinical relevance, as the microdialysis technique used is identical to clinical routine, the treatment regimen is translationally sound and the model is unaffected by potential neuroprotective properties of anaesthetics.

### The ET-1 model as ischaemia-reperfusion model

Under clinical conditions, infarct size has been shown to decrease in stroke patients treated with CsA only after successful recanalisation^18,19^. Hence, an animal model with *in vivo* testing of the neuroprotective properties of CsA should possess both ischaemia and reperfusion properties.

In the present model, we achieved a transient constriction of the proximal middle cerebral artery through stereotaxic application of ET-1, as previously described^20–22^. Contrary to the widespread standard MCAO model, the ET-1 model allows the rats to be awake and freely moving and avoids the potential confounding effects of anaesthetics. Combined with cerebral microdialysis, this enables continuous monitoring of cerebral energy metabolism during interventions under translational conditions.

### CsA as a neuroprotective agent

The neuroprotective effect of CsA, a drug otherwise used for immunosuppression purposes, is believed to be exerted through inhibition of the cyclophilin D-mediated activation of the mitochondrial permeability transition pore (mPTP). The mPTP is activated under various pathophysiological conditions such as trauma, infection and cerebral ischaemia, inducing mitochondrial dysfunction and cell death^27–29^.

Previous experimental and clinical studies of CsA have utilised the Sandimmune formulation. This drug is known to be associated with severe anaphylactic complications, probably since CsA is administered in the polyoxyethylated castor oil base Cremophor EL^30,31^. The present study was conducted with a lipid-free emulsion (CicloMulsion) of CsA (NeuroSTAT) with fewer adverse reactions^32^.

### Dosing, timing and route of administration

There is an overlap of reported optimal treatment effect on one side and toxicity on the other, concerning dose, time-window and route of administration in CsA treatment for neuroprotective purposes^33^. The intravenous route of administration is translationally preferable, and the optimal dose ranges from 10 mg/kg to 18.75 mg/kg, with reported side-effects at the higher dose^34^. For single dose i.v. administration, 15 mg/kg, 0.5 mg/ml CsA was thus chosen as a tolerable and efficient dose in the present study. The timing aspect is also important, with positive results occurring in pre-ischaemia treatment regimens and up to 6–24 hour post-insult^35,36^. Pre-reperfusion treatment, as opposed to treatment prior to the ischaemic event itself, is clinically plausible in patients selected for thrombolysis and thrombectomy as well as in patients with the stroke subtype subarachnoid haemorrhage with a high risk of delayed cerebral ischaemia.

### The biochemical pattern during ischaemia-reperfusion and CsA treatment

In the present study, the levels of interstitial glucose decreased with reduced cerebral blood flow and then increased to above baseline during reperfusion. This pattern is compatible with cerebral ischaemia-reperfusion^37^.

The LPR increased five-fold during ischaemia. Although both treatment and placebo groups subsequently slowly returned toward baseline, the LPR level of the CsA group decreased significantly faster after reperfusion (Fig. 2D).

LPR reflects the cytoplasmic redox state, and compromised energy metabolism will cause an immediate increase in LPR^38^. Different biochemical patterns of LPR have been observed under various pathological metabolic conditions:

1. *Cerebral ischaemia*. Decreased blood flow yields a pattern characterised by an elevated LPR with simultaneous reduced tissue levels of glucose and pyruvate^21,39,40^.
2. *Excessive cerebral energy utilisation*. When energy demands exceed the mitochondrial capacity for oxidative metabolism e.g. during generalised epileptic seizures, the biochemical pattern is characterised by increased LPR, increased pyruvate and decreased cerebral glucose concentration^41,42^.
3. *Hypoxia*. In pronounced arterial hypoxia, LPR and tissue pyruvate increase and – as cerebral blood flow is increased – so does the tissue concentration of glucose^43^.
4. *Mitochondrial dysfunction*. In experimentally induced mitochondrial dysfunction, the metabolic pattern is characterised by a large increase in tissue lactate concentration together with a normal or moderately increased pyruvate level^3,4,44,45^. Similar patterns have been described in clinical situations with supposed mitochondrial dysfunction e.g. post-ischaemic recirculation and bacterial meningitis^5,6,46^.

As we did not observe seizures or hypoxic hypoxia, the LPR changes we observed in both groups are interpreted as transient ischaemia with subsequent mitochondrial dysfunction. This is supported by previous studies documenting mitochondrial dysfunction cerebral ischaemia-reperfusion injury^1,2^. The fact that LPR after reperfusion is significantly lower in animals treated with CsA (NeuroSTAT 15 mg/kg i.v.) suggests mitochondrial stabilisation. The ameliorating effect of CsA on energy metabolism is further supported by the reduction in 24-hour infarct volumes.

### Limitations

Although our animals treated with CsA showed a marked (albeit smaller) striatal infarct upon histologic examination, the biochemical results nearly normalised after reperfusion. A similar finding has been described in patients with malignant cerebral infarct treated with hemicraniectomy^5^. To further explore this aspect of post-ischaemia metabolism, perfusion fluid labelling coupled with metabolomic analysis may be useful^47^.

The short duration from lesion and treatment to sacrifice (24 hours) may have reduced infarct size differences^48^, but it was sufficient for the study aim and in agreement with refinement guidelines of animal study ethics.

CsA only passes the intact blood-brain barrier in small amounts, and disruption of the blood-brain barrier is crucial to treatment efficacy^49^. This problem, bypassed in the current study by the intracerebral double guide insertion inherent to the model, may be one of the remaining obstacles in efficient clinical treatment strategies.

## Conclusions

This study found that CsA (NeuroSTAT) reduced infarct volumes in the ET-1 rat model of focal transient cerebral ischaemia. This correlated with improved oxidative metabolism, as shown by decreased post-ischaemic LPR on intracerebral microdialysis. The results suggest that CsA may be neuroprotective in selected cerebrovascular patient groups and that the ET-I rat model may be useful in pre-clinical trials of putative neuroprotective substances. Further, routine cerebral microdialysis should be considered in clinical trials of neuroprotective treatment in cerebrovascular disease, in addition to evaluations of the short- and long-term outcome.

## Data Availability

The datasets generated and analysed during the current study are available from the corresponding author on reasonable request.
